# Association of Timing of Plasma Transfusion With Adverse Maternal Outcomes in Women With Persistent Postpartum Hemorrhage

**DOI:** 10.1001/jamanetworkopen.2019.15628

**Published:** 2019-11-15

**Authors:** Dacia D. C. A. Henriquez, Camila Caram-Deelder, Saskia le Cessie, Joost J. Zwart, Jos J. M. van Roosmalen, Jeroen C. J. Eikenboom, Cynthia So-Osman, Leo M. G. van de Watering, Jaap Jan Zwaginga, Ankie W. M. M. Koopman-van Gemert, Kitty W. M. Bloemenkamp, Johanna G. van der Bom

**Affiliations:** 1 Department of Obstetrics, Leiden University Medical Center, Leiden, the Netherlands; 2 Jon J van Rood Center for Clinical Transfusion Research, Sanquin-Leiden University Medical Center, Leiden, the Netherlands; 3 Department of Clinical Epidemiology, Leiden University Medical Center, Leiden, the Netherlands; 4 Department of Biomedical Data Sciences, Leiden University Medical Center, Leiden, the Netherlands; 5 Department of Obstetrics and Gynecology, Deventer Hospital, Deventer, the Netherlands; 6 Athena Institute, Vrije Universiteit, Amsterdam, the Netherlands; 7 Division of Thrombosis and Hemostasis, Department of Internal Medicine, Leiden University Medical Center, Leiden, the Netherlands; 8 Sanquin Blood Bank, Unit Transfusion Medicine, Leiden, the Netherlands; 9 Department of Immunohematology and Blood Transfusion, Leiden University Medical Center, Leiden, the Netherlands; 10 Department of Anesthesiology, Albert Schweitzer Hospital, Dordrecht, the Netherlands; 11 Department of Obstetrics, Birth Center Wilhelmina’s Children Hospital, Division Woman and Baby, University Medical Centre Utrecht, Utrecht, the Netherlands

## Abstract

**Importance:**

Early plasma transfusion for women with severe postpartum hemorrhage (PPH) is recommended to prevent coagulopathy. However, there is no comparative, quantitative evidence on the association of early plasma transfusion with maternal outcomes.

**Objective:**

To compare the incidence of adverse maternal outcomes among women who received plasma during the first 60 minutes of persistent PPH vs women who did not receive plasma for similarly severe persistent PPH.

**Design, Setting, and Participants:**

This multicenter cohort study used a consecutive sample of women with persistent PPH, defined as PPH refractory to first-line measures to control bleeding, between January 1, 2011, and January 1, 2013. Time-dependent propensity score matching was used to select women who received plasma during the first 60 minutes of persistent PPH and match each of them with a woman who had shown the same severity and received the same treatment of PPH but who had not received plasma at the moment of matching. Transfusions were not guided by coagulation tests. Statistical analysis was performed from June 2018 to June 2019.

**Exposures:**

Transfusion of plasma during the first 60 minutes of persistent PPH vs no or later plasma transfusion.

**Main Outcomes and Measures:**

Incidence of adverse maternal outcomes, defined as a composite of death, hysterectomy, or arterial embolization.

**Results:**

This study included 1216 women (mean [SD] age, 31.6 [5.0] years) with persistent PPH, of whom 932 (76.6%) delivered vaginally and 780 (64.1%) had PPH caused by uterine atony. Seven women (0.6%) died because of PPH, 62 women (5.1%) had a hysterectomy, and 159 women (13.1%) had arterial embolizations. Among women who received plasma during the first 60 minutes of persistent PPH, 114 women could be matched with a comparable woman who had not received plasma at the moment of matching. The incidence of adverse maternal outcomes was similar between the women, with adverse outcomes recorded in 24 women (21.2%) who received early plasma transfusion and 23 women (19.9%) who did not receive early plasma transfusion (odds ratio, 1.09; 95% CI, 0.57-2.09). Results of sensitivity analyses were comparable to the primary results.

**Conclusions and Relevance:**

In this cohort study, initiation of plasma transfusion during the first 60 minutes of persistent PPH was not associated with adverse maternal outcomes compared with no or later plasma transfusion, independent of severity of PPH.

## Key Points

**Question:**

Is plasma transfusion within the first 60 minutes of persistent postpartum hemorrhage (PPH) associated with incidence of maternal adverse outcomes?

**Findings:**

In this cohort study of 114 propensity score–matched women with persistent PPH, plasma transfusion within the first 60 minutes of persistent PPH was not associated with incidence of maternal adverse outcomes compared with no or later plasma transfusion, independent of severity of PPH at the time of plasma transfusion.

**Meaning:**

These findings do not support the theory that early plasma transfusion in women with persistent PPH is better than no or later plasma transfusion.

## Teaser

This cohort study examines the association of timing of receipt of plasma transfusions among women experiencing persistent postpartum hemorrhage with adverse maternal outcomes.

## Introduction

Obstetric hemorrhage accounts for 27% of all maternal deaths.^1^ In high-resource settings, maternal death due to postpartum hemorrhage (PPH) has become uncommon, but PPH remains an important cause of severe maternal morbidity.^2,3,4,5,6,7^

Women with persistent PPH are at risk of developing coagulopathy due to depletion of coagulation factors and platelets.^8,9,10,11,12^ Coagulopathy can eventually lead to worse maternal outcomes. Timely transfusion of plasma may prevent coagulopathy and thereby improve maternal outcomes.

Results from a 2015 study^13^ among patients with trauma suggest that formulaic plasma transfusion, comprising a fixed ratio of plasma to red blood cells (RBCs), is associated with better outcomes. Whether such transfusion strategies are also associated with better outcomes among women with persistent PPH is not clear. Some studies have suggested that early and aggressive plasma transfusion has a positive association with outcomes in women with PPH.^14,15,16,17,18,19^ However, a 2017 study^20^ suggested that women with persistent PPH have better outcomes when plasma transfusion is postponed or even avoided. Uncertainty about the outcomes associated with plasma transfusion among women with persistent PPH can lead to significant variation in clinical practice. This variation in practice, along with careful documentation of confounding factors, enables the use of routinely collected clinical data to compare outcomes among women treated according to different treatment strategies.

The aim of this study was to assess whether early plasma transfusion is associated with improved maternal outcomes in women with persistent PPH. Our hypothesis was that initiation of plasma transfusion during the first 60 minutes of persistent PPH would be associated with fewer adverse maternal outcomes, defined as maternal death, hysterectomy, or arterial embolization compared with women who received no or later plasma transfusion.

## Methods

Approval was obtained from the Medical Ethics Research Committee of the Leiden University Medical Center and from the institutional review board of each study center, and a waiver of informed consent was granted because the study used deidentified data. The study was registered in the Netherlands Trial Register^21^ and reported according to the Strengthening the Reporting of Observational Studies in Epidemiology (STROBE) reporting guideline.

### Study Design and Population

The transfusion strategies in women during major obstetric hemorrhage (TeMpOH-1) study^21^ was a multicenter, retrospective cohort study in the Netherlands that included consecutive women who had received 4 or more units of RBCs or a multicomponent blood transfusion within 24 hours after giving birth because of severe PPH (ie, ≥1000 mL blood loss) from January 1, 2011, to January 1, 2013. A multicomponent blood transfusion was defined as transfusion of at least 1 unit of packed RBCs in combination with plasma or platelet concentrates. We selected women from transfusion databases and birth registries in 61 participating hospitals.

From this cohort, we identified women with persistent PPH, defined as PPH with at least 1000 mL of blood loss refractory to first-line interventions to control bleeding.^8,22^ First-line interventions depended on the cause of bleeding, as previously described (eTable 1 in the Supplement).^23^ We regarded the time of initiation of the first-line intervention to stop PPH as the moment of diagnosis of persistent PPH, under the assumption that refractoriness to first-line treatment would become evident shortly after initiation of this therapy. Women were followed up from onset until cessation of PPH.

We excluded women with unknown timing of initiation of plasma transfusion. We also excluded women with initiation of plasma transfusion for any reason other than correcting coagulopathy secondary to PPH (ie, comorbidity).

### Data Collection

Trained medical students and research nurses uniformly performed comprehensive health record reviews. From routinely collected medical information, we reconstructed the treatment course of every woman with PPH. We checked all data for completeness and inconsistencies and repeated on-site health record review as necessary. Data included comorbidity; mode of birth; primary cause of hemorrhage; consecutive estimates of blood loss and time of estimations; blood pressure, heart rate, and time of measurements; volume of crystalloids and colloids for fluid resuscitation; time of transfusions of packed RBCs, plasma, and platelets; and time of obstetric, radiological, and hemostatic interventions to stop bleeding.

### Fresh Frozen Plasma Transfusion

Women with plasma transfusions received 1 or more units of fresh frozen plasma during the treatment of persistent PPH. Transfusion of plasma was not guided by coagulation tests. The time to plasma transfusion was defined as the interval between the moment of diagnosis of persistent PPH and administration of the first unit of plasma.

Previous studies on hemostatic interventions to treat coagulopathy in pregnant and nonpregnant patients with major hemorrhage showed beneficial associations of these interventions when initiated early after the start of hemorrhage, specifically within 3 hours.^24,25^ Therefore, we examined the association of plasma transfusion during the first 60 minutes of persistent PPH with maternal outcomes.

### Outcome

The outcome was the incidence of adverse maternal outcomes, defined as a composite of death, hysterectomy, or arterial embolization to control bleeding. The end of bleeding was defined as the time of the final recorded measurement of blood loss or the time of the last obstetric intervention to stop bleeding.

In the Netherlands, uterine or internal iliac artery embolization is performed before resorting to hysterectomy, if the woman’s hemodynamic condition is stable enough to perform this procedure. During our study, 83.6% of the hospitals had this treatment modality available 24 hours per day, 7 days per week, and 92.5% of our study population gave birth in 1 of these hospitals. If a hospital does not have this treatment modality available, it is common practice to transfer the woman with PPH to a nearby hospital with embolization facilities. Embolization has almost completely substituted ligation of uterine or internal iliac arteries in the Netherlands, and in our study, ligation of arteries was performed in 0.8% of women with persistent PPH.

### Statistical Analysis

Women with more severe PPH are more likely to receive early plasma transfusion, which confounds the association of early plasma transfusion with maternal outcomes. We used time-dependent propensity score matching to ensure that the contrasted groups were similar in terms of severity of hemorrhage and other treatments for PPH.^26,27,28,29,30,31^ First, we calculated the predicted probability to receive early plasma transfusion for all women in the cohort. Second, we selected pairs of women with the same probability for receiving plasma transfusion. These pairs consisted of one woman who received early plasma transfusion and another woman who did not. Third, we compared the matched groups.

#### Propensity Scores

The propensity score reflects the estimated probability of initiation of plasma transfusion in women with persistent PPH, given the observed characteristics of the women at the time of initiation of plasma transfusion.^28,29^ We calculated a propensity score for every woman with persistent PPH by using a multivariable Cox proportional hazards model. The outcome variable in this model was time to plasma transfusion, and the linear predictor at any given minute from diagnosing persistent PPH was used as the propensity score. In women with initiation of plasma transfusion before diagnosing persistent PPH (ie, women with placental abruption), we considered the time of diagnosing persistent PPH as the time of initiating plasma transfusion.

We included baseline and time-dependent covariates associated with initiation of plasma transfusion and maternal outcome in a Cox model to calculate propensity scores. Selection of these potentially confounding variables was based on clinical reasoning and prior knowledge.^7,8,32,33,34,35,36^ The baseline covariates were mode of birth (ie, vaginal or cesarean), cause of hemorrhage (ie, uterine atony, retained placenta, abnormally invasive placenta, or other), preeclampsia (yes or no), and volume of crystalloids and colloids for fluid resuscitation (continuous variable). We included the following time-dependent variables: estimated volume of blood loss (continuous variable), bleeding rate (continuous variable), hemorrhagic shock (yes or no), oxytocin infusion (yes or no), misoprostol (yes or no), ergometrine (yes or no), the prostaglandin E2 analogue sulprostone (yes or no), manual removal of placenta (yes or no), exploration of uterine cavity and genital tract with anesthesia (yes or no), intrauterine balloon tamponade (yes or no), tranexamic acid (yes or no), fibrinogen concentrate (yes or no), recombinant factor VIIa (yes or no), packed RBCs transfusion (categorized as 0, 1, 2, 3, or ≥4 units), and platelet transfusion (yes or no). Additional information on handling of the time-dependent covariates in statistical analyses is provided in eTable 2 in the Supplement.

#### Matching

**Figure 1.**
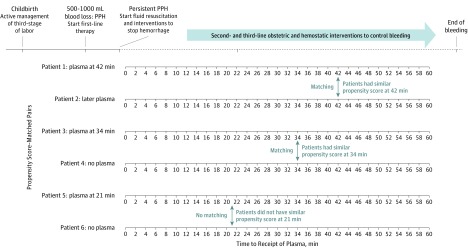
Time-Dependent Propensity Score Matching of Women With Persistent Postpartum Hemorrhage (PPH) Propensity score is the probability of plasma transfusion at a specific time point, given the woman’s observed characteristics at that time point.

We applied a 1:1 nearest-neighbor risk-set matching algorithm on the propensity score without replacement, with a maximum caliper width of 0.1 of the SD of the logit of the propensity score.^37,38,39,40^ In this way, we sequentially matched every woman with persistent PPH in whom plasma transfusion was initiated at any given time point (0-60 minutes after diagnosis of persistent PPH) to a woman with similar propensity score in whom plasma transfusion was not initiated before or at that same time point (Figure 1). In this matched counterpart, plasma transfusion may have been initiated at a later time during PPH. After cessation of PPH or after reaching an endpoint (ie, arterial embolization, hysterectomy, or death), a woman was no longer considered at risk for plasma transfusion for correction of coagulopathy during ongoing hemorrhage.

Missing covariate data were imputed by using multiple imputation.^41,42,43^ We included all confounding variables, outcome variables, and parameters associated with the missing variables as predictive variables in the imputation models and generated 10 imputed data sets. We tested our Cox model for nonproportional hazards by adding interactions with time.

In each imputed data set, we estimated the propensity score for initiation of plasma transfusion for each woman with persistent PPH. We performed a time-dependent propensity score matching within each of these imputed data sets, and then we pooled the effect estimates by averaging them according to the Rubin’s rule.^44,45,46^

After matching, we performed a check of the balance between the confounding variables to ensure our propensity score model was specified correctly. To this end, we calculated the standardized differences in the confounding variables between the women with plasma transfusion during the first 60 minutes of persistent PPH and the women with no or later plasma transfusion in our matched cohort.^47,48,49,50^ Absolute standardized differences less than 10% are generally considered a good balance of the observed confounding variables.^28,51,52^

#### Main and Sensitivity Analyses

We used logistic regression to assess the adjusted association of plasma transfusion during the first 60 minutes of persistent PPH with adverse maternal outcomes; the composite maternal outcome was the dependent variable, and time of plasma transfusion (ie, early vs no or later transfusion) was the independent variable. We used robust SEs to calculate 95% CIs.

We performed several sensitivity analyses to assess the robustness of our results and to assess whether our effect estimate was influenced by women with plasma at a later time point in our comparison group. First, we performed sensitivity analyses with initiation of plasma transfusion during the first 120 and 180 minutes of persistent PPH because a potential beneficial effect of correction of coagulopathy has been previously described within the first 3 hours after the onset of hemorrhage in obstetric and nonobstetric populations.^24,25^

Second, we performed sensitivity analyses by excluding pairs of women with a crossover of the woman initially without plasma to treatment with plasma shortly after matching. These analyses were performed with a restriction of 15, 30, 45, and 60 minutes on the time interval of switching from no plasma to plasma treatment. For example, if a woman treated with plasma at 50 minutes was matched to a women without plasma until 50 minutes but with initiation of plasma at 64 minutes, we excluded this pair in the sensitivity analysis for no crossover within 15 minutes.

Third, we performed sensitivity analyses by excluding pairs of women with a crossover of the woman initially without plasma to treatment with plasma while still being within the first 60 minutes of persistent PPH. For example, if a woman treated with plasma at 30 minutes was matched to a woman without plasma at 30 minutes but with initiation of plasma at 55 minutes, we excluded the pair from this sensitivity analysis.

## Results

### Population

**Figure 2.**
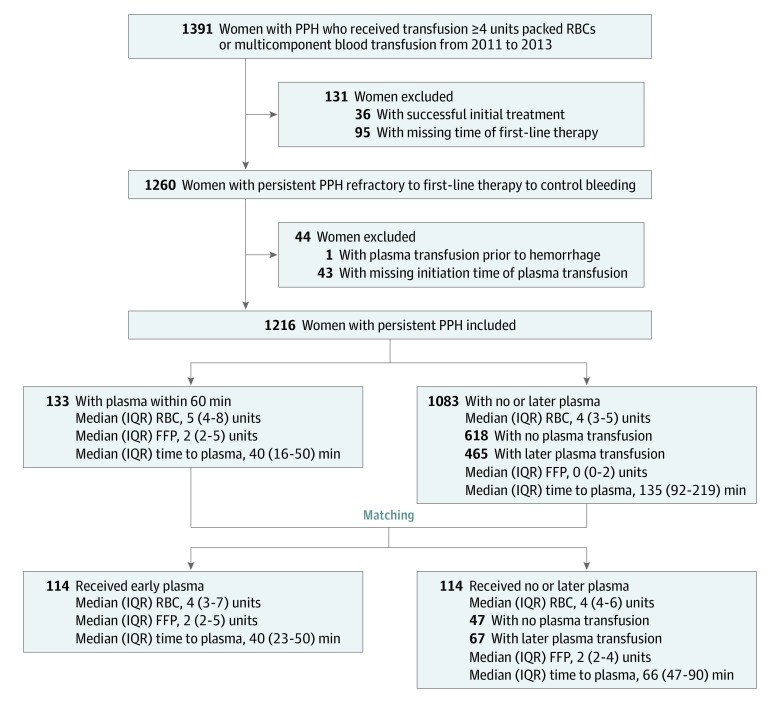
Derivation of Study Population FFP indicates fresh frozen plasma; IQR, interquartile range; PPH, postpartum hemorrhage; and RBC, red blood cell.

The cohort included 1391 women with PPH who received 4 or more units of packed RBCs or a multicomponent blood transfusion within 24 hours after birth (Figure 2). Of these women, we classified 1260 (90.6%) as having persistent PPH. We excluded 43 women with persistent PPH because of unknown time of initiation of plasma transfusion and 1 woman in whom plasma transfusion had been started before birth because of leukemia instead of obstetric hemorrhage. Our final cohort included 1216 women (mean [SD] age, 31.6 [5.0] years). Seven women (0.6%) died because of PPH, 62 women (5.1%) had a hysterectomy, and 159 women (13.1%) had arterial embolizations.

A total of 598 women (49.2%) received plasma during ongoing PPH. Among women in the no or later plasma transfusion group, 618 women (57.1%) did not receive plasma and 465 women (42.9%) received plasma at a later time after matching. Median (interquartile range [IQR]) time to initiation of plasma transfusion was 105 (65-196) minutes. Overall, plasma transfusion was initiated during the first 60 minutes of persistent PPH in 133 women (10.9%), during the first 120 minutes in 338 women (27.8%), and during the first 180 minutes in 433 women (35.6%).

Baseline and time-dependent characteristics of women with early plasma transfusion vs no or later plasma transfusion are presented in Table 1. We imputed missing data on volume of fluid resuscitation (16.0%) and hemorrhagic shock at moment of diagnosing persistent PPH (34.9%). For this latter time-dependent confounding variable, more data (ie, measured blood pressures and heart rates) became available for an increasing proportion of women with progression of the PPH. An adverse maternal outcome was observed in 30 women (22.6%) with plasma transfusion during the first 60 minutes of persistent PPH and in 175 women (16.2%) with no or later plasma transfusions (odds ratio, 1.51; 95% CI, 0.98-2.34) (Table 2).

**Table 1.** Characteristics of Women With Persistent PPH in the Total Cohort and the Propensity Score–Matched Cohort Stratified by Plasma Transfusion Strategy

Characteristic	Overall Cohort at Moment of Diagnosing Persistent PPH		Propensity Score–Matched Cohort at Moment of Matching		
	Women, No. (%)		Women, Pooled Average, No. (%)		Standardized Difference After Propensity Score Matching, %
	No or Later Plasma Transfusion (n = 1083)^a^	Plasma Transfusion Within 60 Minutes (n = 133)	No or Later Plasma Transfusion (n = 114)^a^^,^^b^	Plasma Transfusion Within 60 Minutes (n = 114)^b^^,^^c^	
Mode of birth					
Vaginal	846 (78.1)	86 (64.7)	82 (72.0)	76 (66.7)	2.9
Cesarean	231 (21.3)	47 (35.3)	32 (28.0)	38 (33.3)	
Unknown	6 (0.6)	0	NA	NA	
Cause of hemorrhage^d^					
Uterine atony	701 (64.7)	79 (59.4)	70 (60.9)	69 (60.8)	[Reference]
Retained placenta	188 (17.4)	24 (18.0)	24 (20.9)	20 (17.4)	0.8
Abnormally invasive placenta	93 (8.6)	12 (9.0)	7 (6.3)	11 (9.6)	0.6
Other^e^	101 (9.3)	18 (13.5)	14 (11.9)	14 (12.2)	5.4
Preeclampsia	107 (9.9)	19 (14.3)	9 (7.9)	17 (15.1)	3.1
Fluid resuscitation with crystalloids and colloids, L^f^					2.0
≤2	266 (24.6)	32 (24.1)	27 (23.7)	33 (28.8)	
>2 to ≤4	438 (40.4)	46 (34.6)	61 (53.2)	55 (48.5)	
>4	211 (19.5)	29 (21.8)	26 (23.1)	26 (22.7)	
Unknown	168 (15.5)	26 (19.5)	NA	NA	
Volume of blood loss, L^f^					
≤1	605 (55.9)	43 (32.3)	8 (7.4)	2 (1.8)	1.6
>1 to ≤2	349 (32.2)	45 (33.8)	34 (29.5)	35 (30.3)	
>2	129 (11.9)	45 (33.8)	72 (63.1)	78 (68.0)	
Bleeding rate, L/h^f^					
≤1	576 (53.2)	64 (48.1)	57 (49.8)	44 (38.4)	4.0
>1 to ≤2	231 (21.3)	33 (24.8)	33 (28.5)	44 (38.3)	
>2	276 (25.5)	36 (27.1)	25 (21.6)	27 (23.3)	
Hemorrhagic shock					
No	378 (34.9)	56 (42.1)	47 (41.5)	59 (51.4)	9.5
Yes	303 (28.0)	55 (41.4)	67 (58.5)	56 (48.6)	
Unknown	402 (37.1)	22 (16.5)	NA	NA	
Obstetric interventions					
Oxytocin infusion	422 (39.0)	34 (25.6)	45 (39.1)	53 (46.1)	1.3
Misoprostol	153 (14.1)	12 (9.0)	21 (18.7)	19 (16.6)	6.9
Ergometrine	23 (2.1)	1 (0.8)	11 (9.5)	4 (3.4)	2.6
Sulprostone	59 (5.4)	35 (26.3)	62 (54.3)	60 (52.5)	5.1
Manual removal of placenta	160 (14.8)	37 (27.8)	43 (37.2)	41 (35.5)	5.7
Exploration of uterine cavity and genital tract	77 (7.1)	28 (21.1)	57 (49.6)	57 (50.3)	7.6
Intrauterine balloon tamponade	8 (0.7)	1 (0.8)	18 (15.3)	21 (18.2)	3.2
Hemostatic interventions^g^					
Tranexamic acid	19 (1.8)	17 (12.8)	39 (34.1)	39 (33.8)	2.0
Fibrinogen concentrate	5 (0.5)	2 (1.2)	4 (3.1)	5 (4.4)	2.5
Transfusion^d^					
Packed red blood cells, units					
0	1050 (97.0)	97 (72.9)	25 (21.9)	26 (22.8)	[Reference]
1	14 (1.3)	12 (9.0)	20 (17.8)	23 (20.1)	5.8
2	11 (1.0)	13 (9.8)	41 (35.8)	36 (31.2)	5.1
3	4 (0.4)	3 (2.3)	14 (12.5)	19 (16.5)	4.6
≥4	4 (0.4)	8 (6.0)	14 (12.0)	11 (9.5)	9.4
≥1 Unit of platelets	2 (0.2)	4 (3.0)	2 (1.5)	4 (3.5)	0.7

Abbreviations: NA, not applicable; PPH, postpartum hemorrhage.

^a^ Includes women with no FFP transfusion and women with FFP transfusion at a later time during PPH.

^b^ The proportion of women who have undergone a time-dependent intervention increases during the course of PPH, as an increasing amount of interventions will be performed in a single woman until cessation of the hemorrhage.

^c^ Numbers of women and percentages are means derived from 10 imputed databases, and numbers of women were rounded to the nearest integer. Therefore, they may exceed the total number of women or a proportion of 1, and the same number of women may correspond to different proportions.

^d^ Covariate entered as a categorical variable in the propensity score model.

^e^ Includes genital tract trauma, placenta previa, placental abruption, and congenital or acquired coagulation disorders.

^f^ Covariate entered as a continuous variable in the propensity score model.

^g^ Recombinant factor VIIa was not given to any woman prior to diagnosing persistent PPH or matching.

**Table 2.** Outcomes of Women With Persistent PPH in the Total Cohort and the Propensity Score–Matched Cohort Stratified by Plasma Transfusion Strategy

Outcome	Unadjusted Analyses			Propensity Score–Matched Analyses^a^		
	Women With Outcome, No./Total No. (%)		OR (95% CI)	Women With Outcome, No./Total No. (%)		OR (95% CI)
	No Plasma Transfusion^b^	Plasma Transfusion		No Plasma Transfusion^b^^,^^c^	Plasma Transfusion^b^	
**Plasma Within 60 min**						
Composite	175/1083 (16.2)	30/133 (22.6)	1.51 (0.98-2.34)	23/114 (19.9)	24/114 (21.2)	1.09 (0.57-2.09)
Mortality	5/1083 (0.5)	2/133 (1.5)		2/114 (1.3)	2/114 (1.8)	
Hysterectomy	50/1083 (4.6)	12/133 (9.0)		10/114 (8.3)	10/114 (8.9)	
Arterial embolization	137/1083 (12.7)	22/133 (16.5)		16/114 (13.9)	18/114 (15.8)	
**Plasma Within 120 min**						
Composite	128/878 (14.6)	77/338 (22.8)	1.73 (1.26-2.37)	59/283 (21.0)	59/283 (21.0)	1.00 (0.67-1.51)
Mortality	3/878 (0.3)	4/338 (1.2)		2/283 (0.8)	4/283 (1.4)	
Hysterectomy	37/878 (4.2)	25/338 (7.4)		19/283 (6.7)	20/283 (7.2)	
Arterial embolization	99/878 (11.3)	60/338 (17.8)		47/283 (16.5)	45/283 (15.9)	
**Plasma Within 180 min**						
Composite	95/783 (12.1)	110/433 (25.4)	2.47 (1.82-3.35)	80/348 (23.0)	77/348 (22.2)	0.96 (0.67-1.37)
Mortality	3/783 (0.4)	4/433 (0.9)		4/348 (1.0)	4/348 (1.1)	
Hysterectomy	28/783 (3.6)	34/433 (7.9)		23/348 (6.5)	27/348 (7.7)	
Arterial embolization	73/783 (9.3)	86/433 (19.9)		64/348 (18.5)	58/348 (16.6)	

Abbrevations: OR, odds ratio; PPH, postpartum hemorrhage.

^a^ Adjusted for all variables included in the propensity score.

^b^ Includes women without plasma transfusion and women with plasma transfusion at a later time during PPH.

^c^ Numbers of women and percentages are pooled averages derived from 10 imputed databases, and numbers of women were rounded to the nearest integer. Therefore, they may exceed the total number of women or a proportion of 1, and the same number of women may correspond to different proportions.

### Time-Dependent Propensity Score–Matched Population

The number of matched pairs of women with plasma transfusion during the first 60 minutes and women with no or later plasma transfusion fluctuated across the 10 imputed data sets. We found a pooled average of 114 matches of women with plasma transfusion during the first 60 minutes and women with no plasma or plasma transfusion at a later time during persistent PPH. Nineteen women with plasma transfusion during the first 60 minutes had no match on propensity score (Table 1). Median (IQR) time to plasma transfusion in women with plasma transfusion during the first 60 minutes was 40 (16-50) minutes. Of their matched counterparts, 47 women (41.2%) did not receive plasma during PPH and 67 women (58.8%) received plasma at a later time during PPH, with a median (IQR) time to plasma transfusion of 66 (47-90) minutes in these 67 women. Across the 10 imputed data sets, we included a pooled average of 29 women twice in this matched cohort: first as a woman with no or later plasma transfusion and later as a woman with plasma transfusion during the first 60 minutes.

### Outcomes in Adjusted Analyses

**Figure 3.**
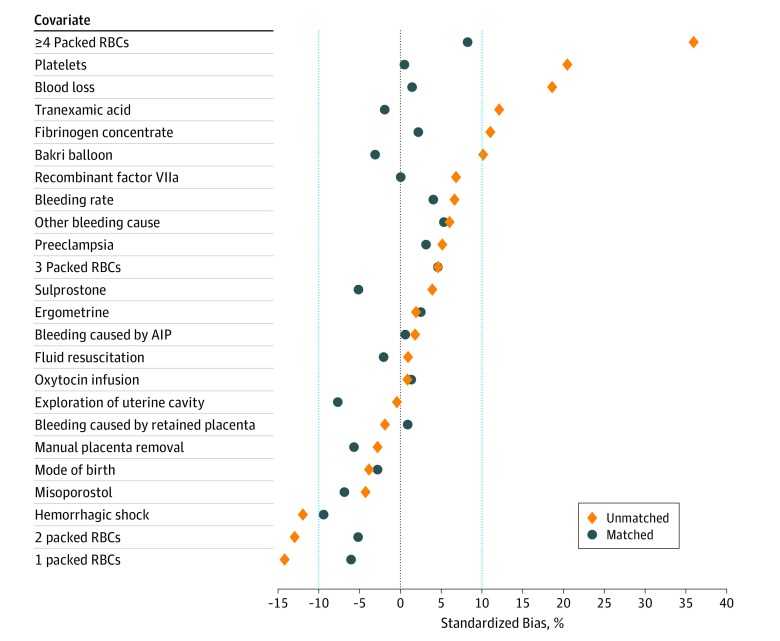
Balance of Covariate Values After Time-Dependent Propensity Score Matching of Women With Persistent Postpartum Hemorrhage AIP indicates abnormally invasive placenta and RBC, red blood cell.

The distribution of baseline and time-dependent covariates in the matched cohort were well balanced between women with plasma transfusion during the first 60 minutes and women with no or later plasma transfusion (Figure 3 and Table 2). In the matched cohort, we observed a pooled average of 24 adverse maternal outcomes (21.2%) in women with plasma transfusion within 60 minutes vs 23 adverse maternal outcomes (19.9%) in women with no or later plasma transfusion (odds ratio, 1.09; 95% CI, 0.57-2.09).

### Sensitivity Analyses

Unadjusted and adjusted sensitivity analyses in women with plasma transfusion within 120 minutes and within 180 minutes vs no or later plasma transfusion within these intervals yielded similar results as the primary analysis (Table 2) (eTable 3 and eTable 4 in the Supplement). In the sensitivity analyses excluding pairs of women in which a woman crossed over from no or later plasma to plasma transfusion 15, 30, 45, or 60 minutes after matching, we also found effect estimates comparable to our main analysis (eTable 5 in the Supplement). In the sensitivity analysis excluding 29 pairs of women because of crossover from no or later plasma to plasma transfusion during the first 60 minutes of persistent PPH, the odds ratio was 0.94 (95% CI, 0.43-2.06) for the remaining pairs of women.

## Discussion

In this multicenter, time-dependent propensity score–matched cohort study of women with persistent PPH, empirical, early plasma transfusion was not associated with better maternal outcomes compared with women who received no or later plasma transfusion. Similar results were observed in all sensitivity analyses.

Early plasma transfusion is believed to improve maternal outcomes because it could prevent or treat coagulopathy occurring among women treated for persistent PPH. Studies evaluating the effect of plasma transfusion on outcomes of women with severe PPH are scarce, to our knowledge. Contrary to our findings, a single-center observational study^15^ among 142 women with severe PPH reported a decreased rate of advanced interventions associated with a high ratio of plasma to packed RBCs. In that study, only 41 women received plasma in the management of PPH. Similarly, high ratios of plasma to packed RBCs have been reported to improve maternal outcomes when incorporated within PPH protocols, but whether this improvement could be attributed to the transfusion strategy or to other parts of the protocol is unclear.^17,18^

The observed absence of an effect of early plasma transfusion on maternal outcomes among women with persistent PPH may have several explanations. First, there may have been too few women who developed significant coagulopathy and therefore there was no need to treat or prevent it. This explanation is consistent with findings from studies among women with severe PPH in whom fibrinogen concentrate was administered early during hemorrhage to prevent and correct coagulopathy.^53,54^ In these studies, most women had not developed coagulopathy at the time of administration of fibrinogen, and outcomes did not improve. Yet, in the TeMpOH-1 study cohort,^21^ 26% of women eventually reached a fibrinogen level of less than 200 mg/dL (to convert to micromoles per liter, multiply by 0.0294), and 5% of women reached this level after losing less than 2 L of blood,^55^ which suggests that the absence of coagulopathy in our cohort is not an explanation for our findings.

Second, plasma might not be effective in preventing or treating coagulopathy in women with persistent PPH, or the dose of plasma may have been too low to show a difference. It is conceivable that personalized supplementation of factor concentrates would be a better strategy to prevent adverse outcomes among women with PPH.

Third, 42.9% of the women in the control group were eventually also treated with plasma. Some of these women received plasma relatively shortly after the moment at which they had been matched to their rapidly treated counterpart. If such later plasma was as effective as early administration of plasma, that could explain the observed absence of association of early plasma transfusion with outcomes. Yet, sensitivity analysis among matched pairs without this problem showed similar results, suggesting that this also did not explain our findings.

### Limitations and Strengths

Our findings had some limitations and should be interpreted with caution, as they may also be explained by residual confounding. Women with more severe PPH are more likely to be rapidly treated with plasma than women with less severe hemorrhages. Time-dependent propensity score matching permitted us to balance all measured prognostic factors at any time during PPH, but this technique does not account for the distribution of unknown or unmeasured confounders. Yet, the professionals treating the women with severe PPH in our cohort carefully documented all parameters that are generally considered relevant with respect to the severity and treatment of PPH, to our knowledge. We could not think of any other parameters that might explain the observed absence of association. In addition, our findings may also be explained by random error. The confidence interval around the point estimate included values between 0.57 and 2.09, suggesting that there may be a protective or harmful association of early plasma transfusion with maternal outcomes, in line with the findings of previous studies.^15,16,17,18,19,20^

A strength of our study was the use of persistent PPH, an intuitive and pragmatic definition of severe PPH with easy translation to daily clinical practice, to select women for this analysis.^8,22,36^ In the Netherlands, clinical parameters and the times of interventions are carefully recorded during obstetric emergencies. Thus, we were able to make a detailed reconstruction of the course of PPH, and we had no loss to follow up. In addition, extensive sensitivity analyses showed consistent results.

## Conclusions

This cohort study found that among women with persistent PPH, empirical early plasma transfusion was not associated with maternal deaths, hysterectomies, or arterial embolizations compared with no or later plasma transfusion. Results were carefully adjusted for severity of PPH and time-dependent confounding, but residual confounding cannot be ruled out because of the observational nature of the study design.

Our findings do not suggest that plasma transfusion has no place in the treatment of women with severe PPH. Rather, our study underlines the importance of developing tools to diagnose coagulopathy during persistent PPH. These tools may enable individualization of treatment of women with persistent PPH by identifying women who develop coagulopathy during persistent PPH.

## Notes

Supplement. **eTable 1.** First-Line Interventions to Control Bleeding Stratified by Primary Cause of Postpartum Hemorrhage **eTable 2.** Handling of Time-Dependent Covariates Included in the Propensity Score Model **eTable 3.** Characteristics of Women With Persistent Postpartum Hemorrhage for Sensitivity Analysis at 120 Minutes **eTable 4.** Characteristics of Women With Persistent Postpartum Hemorrhage for Sensitivity Analysis at 180 Minutes **eTable 5.** Sensitivity Analyses Excluding Pairs of Women With Cross-Overs From No or Later Plasma to Plasma Shortly After Matching
