# Supplementary material for: Association of Timing of Plasma Transfusion With Adverse Maternal Outcomes in Women With Persistent Postpartum Hemorrhage
Source: JAMA Netw Open. 2019 Nov 15;2(11):e1915628. doi: 10.1001/jamanetworkopen.2019.15628 (PMC6902812; doi:10.1001/jamanetworkopen.2019.15628)
Supplement: Supplement. — eTable 1. First-Line Interventions to Control Bleeding Stratified by Primary Cause of Postpartum Hemorrhage eTable 2. Handling of Time-Dependent Covariates Included in the Propensity Score Model eTable 3. Characteristics of Women With Persistent Postpartum Hemorrhage for Sensitivity Analysis at 120 Minutes eTable 4. Characteristics of Women With Persistent Postpartum Hemorrhage for Sensitivity Analysis at 180 Minutes eTable 5. Sensitivity Analyses Excluding Pairs of Women With Cross-Overs From No or Later Plasma to Plasma Shortly After Matching [file jamanetwopen-2-e1915628-s001.pdf]

# Supplementary Online Content

Henriquez DDCA, Caram-Deelder C, le Cessie S, et al; for the TeMpOH-1 Research Group. Association of timing of plasma transfusion with adverse maternal outcomes in women with persistent postpartum hemorrhage. *JAMA Netw Open*. 2019;2(11):e1915628. doi:10.1001/jamanetworkopen.2019.15628

**eTable 1.** First-Line Interventions to Control Bleeding Stratified by Primary Cause of Postpartum Hemorrhage

**eTable 2.** Handling of Time-Dependent Covariates Included in the Propensity Score Model

**eTable 3.** Characteristics of Women With Persistent Postpartum Hemorrhage for Sensitivity Analysis at 120 Minutes

**eTable 4.** Characteristics of Women With Persistent Postpartum Hemorrhage for Sensitivity Analysis at 180 Minutes

**eTable 5.** Sensitivity Analyses Excluding Pairs of Women With Cross-Overs From No or Later Plasma to Plasma Shortly After Matching

This supplementary material has been provided by the authors to give readers additional information about their work.

© 2019 Henriquez DDCA et al. *JAMA Network Open.*

**eTable 1.** First-Line Interventions to Control Bleeding Stratified by Primary Cause of Postpartum Hemorrhage

The intervention that was employed first was regarded as the first-line intervention to stop bleeding.

| **Primary cause of postpartum hemorrhage** | **First-line interventions to stop bleeding** |
|---|---|
| Uterine atony | Uterine massage and uterotonic agents (oxytocin, misoprostol, methylergometrine) |
| Retained placenta or placental remnants | Manual removal of placenta, exploration of uterine cavity and uterotonic agents |
| Trauma of birth canal | Surgical repair and uterotonic agents |
| Surgical cause during/after cesarean birth | Surgical repair and uterotonic agents |
| Placental abruption | Cesarean section and uterotonic agents |
| Placenta previa | Cesarean section and uterotonic agents |
| Placenta accreta spectrum | Surgical interventions and uterotonic agents |
| Congenital coagulation disorder or anticoagulant use | Combination of interventions depending on preexistent coagulation disorder |

© 2019 Henriquez DDCA et al. *JAMA Network Open.*

**eTable 2.** Handling of Time-Dependent Covariates Included in the Propensity Score Model

| Variable | Handling in statistical analyses |
|---|---|
| **Volume of blood loss** | Volume of blood loss during postpartum hemorrhage was estimated regularly by weighing all gauzes, cloths and surgical swabs and suction into canisters. We performed linear interpolations between the observed volumes of blood loss to determine the volume of blood loss at any given time during postpartum hemorrhage.<br>This variable was entered as a continuous variable in the propensity score model. |
| **Bleeding rate** | Rate of bleeding was calculated by dividing the volume of blood loss between the two nearest observed measurements by the time between those measurements. The calculated value was carried forward between these two measurements of blood loss volume to determine the bleeding rate at any given time during hemorrhage.<br>This variable was entered as a continuous variable in the propensity score model. |
| **Hemorrhagic shock** | Hemorrhagic shock was considered present with at least one measurement of systolic blood pressure ≤90 mmHg and/or heart rate ≥120 bpm during postpartum hemorrhage. Values were carried forward between measurements of vital parameters to determine whether a woman had experienced hemorrhagic shock at any given time during postpartum hemorrhage. In women with missing vital parameters' values we imputed values every 5 minutes and carried forward between these values.<br>This variable was entered as a dichotomous variable in the propensity score model. |
| **Obstetric interventions:** oxytocin infusion, misoprostol, ergometrine, sulprostone, manual removal of placenta, exploration of uterine cavity, intra-uterine balloon tamponade | The value of every obstetric intervention was 'no' until employment of the intervention. From the time of employment onwards the value was 'yes'.<br>These variables were entered as dichotomous variables in the propensity score model. |
| **Hemostatic interventions:** tranexamic acid, fibrinogen concentrate, recombinant factor VIIa | The value of every hemostatic intervention was 'no' until employment of the intervention. From the time of employment onwards the value was 'yes'.<br>These variables were entered as dichotomous variables in the propensity score model. |
| **Transfusion:** packed red blood cells, platelets | Transfusion of packed red blood cells or platelets was '0' until transfusion of the first unit of these blood products. From the time of transfusion of the first unit the value '1' was carried forward until the time of transfusion of the second unit, and so forth.<br>These variables were entered as categorical variables in the propensity score model. |

© 2019 Henriquez DDCA et al. *JAMA Network Open.*

**eTable 3.** Characteristics of Women With Persistent Postpartum Hemorrhage for Sensitivity Analysis at 120 Minutes

| | **Women, No. (%)** | | | | |
|---|---|---|---|---|---|
| | **Overall cohort** *Characteristics at moment of diagnosing persistent postpartum hemorrhage* | | **Propensity score matched cohort** *Characteristics at moment of matching* | | |
| **Characteristic** | **No or later plasma transfusion[d] (n = 878)** | **Plasma transfusion within 120 minutes (n = 338)** | **No or later plasma transfusion[d,e] (n = 283)** | **Plasma transfusion within 120 minutes[e,f] (n = 283)** | **Standardized difference after propensity score matching (%)** |
| **Mode of birth** | | | | | 2.9 |
| Vaginal | 676 (77.0) | 256 (75.7) | 219 (77.2) | 215 (76.1) | |
| Cesarean | 197 (22.4) | 81 (24.0) | 64 (22.8) | 68 (23.9) | |
| Unknown | 5 (0.6) | 1 (0.3) | - | - | |
| **Cause of hemorrhage[a]** | | | | | |
| Uterine atony | 563 (64.1) | 217 (64.2) | 186 (65.8) | 181 (64.1) | reference |
| Retained placenta | 154 (17.5) | 58 (17.2) | 51 (17.8) | 50 (17.7) | 0.8 |
| Abnormally invasive placenta | 82 (9.3) | 23 (6.8) | 21 (7.5) | 19 (6.7) | 0.6 |
| Other[b] | 79 (9.0) | 40 (11.8) | 25 (8.9) | 33 (11.5) | 5.4 |
| **Preeclampsia** | 87 (9.9) | 39 (11.5) | 27 (9.6) | 29 (10.4) | 3.1 |
| **Fluid resuscitation with crystalloids and colloids[c]** | | | | | 2.0 |
| ≤2L | 229 (26.1) | 69 (20.4) | 70 (24.7) | 70 (24.9) | |
| >2 to ≤4L | 358 (40.8) | 126 (37.3) | 149 (52.7) | 140 (49.6) | |
| >4L | 156 (17.8) | 84 (24.9) | 64 (22.5) | 72 (25.5) | |
| Unknown | 135 (15.4) | 59 (17.5) | - | - | |

© 2019 Henriquez DDCA et al. *JAMA Network Open.*

**eTable 3, *continued*.** Characteristics of Women With Persistent Postpartum Hemorrhage for Sensitivity Analysis at 120 Minutes

| | Women, No. (%) | | | | |
|---|---|---|---|---|---|
| | **Overall cohort** *Characteristics at moment of diagnosing persistent postpartum hemorrhage* | | **Propensity score matched cohort** *Characteristics at moment of matching* | | |
| **Characteristic** | **No or later plasma transfusion[d] (n = 878)** | **Plasma transfusion within 120 minutes (n = 338)** | **No or later plasma transfusion[d,e] (n = 283)** | **Plasma transfusion within 120 minutes[e,f] (n = 283)** | **Standardized difference after propensity score matching (%)** |
| **Volume of blood loss[c]** | | | | | 1.6 |
| ≤1L | 500 (56.9) | 148 (43.8) | 12 (4.3) | 6 (2.1) | |
| >1 to ≤2L | 274 (31.2) | 120 (35.5) | 65 (23.0) | 61 (21.4) | |
| >2L | 104 (11.8) | 70 (20.7) | 206 (72.8) | 216 (76.5) | |
| **Bleeding rate[c]** | | | | | 4.0 |
| ≤1L/h. | 484 (55.1) | 156 (46.2) | 168 (59.5) | 156 (55.1) | |
| >1 to ≤2L/h. | 186 (21.2) | 78 (23.1) | 77 (27.1) | 90 (31.7) | |
| >2L/h. | 208 (23.7) | 104 (30.8) | 38 (13.4) | 38 (13.3) | |
| **Hemorrhagic shock** | | | | | 9.5 |
| No | 314 (35.8) | 120 (35.5) | 128 (45.2) | 137 (48.3) | |
| Yes | 240 (27.3) | 118 (34.9) | 155 (54.8) | 146 (51.7) | |
| Unknown | 324 (36.9) | 100 (29.6) | - | - | |
| **Obstetric interventions** | | | | | |
| Oxytocin infusion | 330 (37.6) | 126 (37.3) | 156 (55.0) | 159 (56.1) | 1.3 |
| Misoprostol | 125 (14.2) | 40 (11.8) | 84 (29.7) | 72 (25.5) | 6.9 |
| Ergometrine | 16 (1.8) | 8 (2.4) | 23 (8.1) | 22 (7.9) | 2.6 |
| Sulprostone | 43 (4.9) | 51 (15.1) | 171 (60.6) | 160 (56.7) | 5.1 |
| Manual removal of placenta | 138 (15.7) | 59 (17.5) | 107 (37.7) | 105 (36.9) | 5.7 |
| Exploration of uterine cavity and genital tract | 60 (6.8) | 45 (13.3) | 162 (57.3) | 155 (54.6) | 7.6 |

© 2019 Henriquez DDCA et al. *JAMA Network Open.*

**eTable 3, *continued*.** Characteristics of Women With Persistent Postpartum Hemorrhage for Sensitivity Analysis at 120 Minutes

| | Women, No. (%) | | | | |
|---|---|---|---|---|---|
| | **Overall cohort** *Characteristics at moment of diagnosing persistent postpartum hemorrhage* | | **Propensity score matched cohort** *Characteristics at moment of matching* | | |
| **Characteristic** | **No or later plasma transfusion (n = 878)[d]** | **Plasma transfusion within 120 minutes (n = 338)** | **No or later plasma transfusion[d,e] (n = 283)** | **Plasma transfusion within 120 minutes[e,f] (n = 283)** | **Standardized difference after propensity score matching (%)** |
| Intra-uterine balloon tamponade (Bakri) | 6 (0.7) | 3 (0.9) | 70 (24.6) | 59 (20.8) | 3.2 |
| **Hemostatic interventions** | | | | | |
| Tranexamic acid | 16 (1.8) | 20 (5.9) | 102 (36.0) | 99 (35.1) | 2.0 |
| Fibrinogen concentrate | 2 (0.2) | 5 (1.5) | 9 (3.1) | 12 (4.1) | 2.5 |
| Recombinant factor VIIa | - | - | - | - | - |
| **Transfusion[a]** | | | | | |
| Packed red blood cells | | | | | |
| 0 | 851 (96.9) | 296 (87.6) | 51 (18.2) | 52 (18.4) | reference |
| 1 | 10 (1.1) | 16 (4.7) | 55 (19.5) | 49 (17.3) | 5.8 |
| 2 | 10 (1.1) | 14 (4.1) | 115 (40.7) | 106 (37.5) | 5.1 |
| 3 | 4 (0.5) | 3 (0.9) | 33 (11.7) | 38 (13.5) | 4.6 |
| ≥4 | 3 (0.3) | 9 (2.7) | 28 (10.0) | 38 (13.4) | 9.4 |
| Platelets | | | | | 0.7 |
| ≥1 | 2 (0.2) | 4 (1.2) | 7 (2.4) | 7 (2.6) | |

[a]Covariate entered as a categorical variable in the propensity score model, with the first category as reference category. [b]includes genital tract trauma, placenta previa, placental abruption and congenital or acquired coagulation disorders. [c]Covariate entered as a continuous variable in the propensity score model. [d]'No or later plasma transfusion' includes women with no plasma transfusion and women with plasma transfusion at a later time point during hemorrage. [e]The proportion of women who have ondergone a time-dependent intervention increases during the course of postpartum hemorrhage, as an increasing amount of interventions will be performed in a single woman until cessation of the hemorrhage.[f]Numbers of women and proportions are averages derived from 10 imputed databases, and numbers of women were rounded to

© 2019 Henriquez DDCA et al. *JAMA Network Open.*

the nearest integer. Therefore, they may exceed the 'total' number of women or a proportion of 1, and the same number of women may correspond to different proportions.

**eTable 4.** Characteristics of Women With Persistent Postpartum Hemorrhage for Sensitivity Analysis at 180 Minutes

| | **Women, No. (%)** | | | | |
|---|---|---|---|---|---|
| | **Overall cohort** *Characteristics at moment of diagnosing persistent postpartum hemorrhage* | | **Propensity score matched cohort** *Characteristics at moment of matching* | | |
| **Characteristic** | **No or later plasma transfusion[d] (n = 783)** | **Plasma transfusion within 180 minutes (n = 433)** | **No or later plasma transfusion[d,e] (n = 348)** | **Plasma transfusion within 180 minutes[e,f] (n = 348)** | **Standardized difference after propensity score matching (%)** |
| **Mode of birth** | | | | | 2.9 |
| Vaginal | 604 (77.1) | 328 (75.8) | 270 (77.6) | 266 (76.4) | |
| Cesarean | 174 (22.2) | 104 (24.0) | 78 (22.4) | 82 (23.6) | |
| Unknown | 5 (0.6) | 1 (0.2) | - | - | |
| **Cause of hemorrhage[a]** | | | | | |
| Uterine atony | 497 (63.5) | 283 (65.4) | 233 (66.9) | 226 (64.8) | reference |
| Retained placenta | 143 (18.3) | 69 (15.9) | 58 (16.6) | 59 (17.0) | 0.8 |
| Abnormally invasive placenta | 68 (8.7) | 37 (8.5) | 28 (8.1) | 29 (8.2) | 0.6 |
| Other[b] | 75 (9.6) | 44 (10.2) | 29 (8.4) | 35 (10.0) | 5.4 |
| **Preeclampsia** | 77 (9.8) | 49 (11.3) | 34 (9.7) | 37 (10.7) | 3.1 |
| **Fluid resuscitation with crystalloids and colloids[c]** | | | | | 2.0 |
| ≤2L | 210 (26.8) | 88 (20.3) | 82 (23.5) | 87 (25.0) | |
| >2 to ≤4L | 312 (39.8) | 172 (39.7) | 178 (51.0) | 177 (50.7) | |
| >4L | 136 (17.4) | 104 (24.0) | 89 (25.5) | 85 (24.3) | |
| Unknown | 125 (16.0) | 69 (15.9) | - | - | |

© 2019 Henriquez DDCA et al. *JAMA Network Open.*

**eTable 4, *continued*.** Characteristics of Women With Persistent Postpartum Hemorrhage for Sensitivity Analysis at 180 Minutes

| | **Women, No. (%)** | | | | |
|---|---|---|---|---|---|
| | **Overall cohort**<br>*Characteristics at moment of diagnosing persistent postpartum hemorrhage* | | **Propensity score matched cohort**<br>*Characteristics at moment of matching* | | |
| **Characteristic** | **No or later plasma transfusion[d] (n = 783)** | **Plasma transfusion within 180 minutes (n = 433)** | **No or later plasma transfusion[d,e] (n = 348)** | **Plasma transfusion within 180 minutes[e,f] (n = 348)** | **Standardized difference after propensity score matching (%)** |
| **Volume of blood loss[c]** | | | | | 1.6 |
| ≤1L | 436 (55.7) | 212 (49.0) | 13 (3.7) | 60 (1.7) | |
| >1 to ≤2L | 251 (32.1) | 143 (33.0) | 74 (21.2) | 74 (21.4) | |
| >2L | 96 (12.3) | 78 (18.0) | 262 (75.1) | 268 (76.9) | |
| **Bleeding rate[c]** | | | | | 4.0 |
| ≤1L/h. | 435 (55.6) | 205 (47.3) | 215 (61.6) | 210 (60.2) | |
| >1 to ≤2L/h. | 167 (21.3) | 97 (22.4) | 91 (26.0) | 101 (29.1) | |
| >2L/h. | 181 (23.1) | 131 (30.3) | 43 (12.4) | 38 (10.8) | |
| **Hemorrhagic shock** | | | | | 9.5 |
| No | 285 (36.4) | 149 (34.4) | 159 (45.5) | 175 (50.3) | |
| Yes | 217 (27.7) | 141 (32.6) | 190 (54.5) | 173 (49.7) | |
| Unknown | 281 (35.9) | 143 (33.0) | - | - | |
| **Obstetric interventions** | | | | | |
| Oxytocin infusion | 296 (37.8) | 160 (37.0) | 199 (57.1) | 201 (57.7) | 1.3 |
| Misoprostol | 107 (13.7) | 58 (13.4) | 112 (32.2) | 101 (29.0) | 6.9 |
| Ergometrine | 14 (1.8) | 10 (2.3) | 26 (7.6) | 29 (8.3) | 2.6 |
| Sulprostone | 36 (4.6) | 58 (13.4) | 217 (62.3) | 209 (59.9) | 5.1 |
| Manual removal of placenta | 134 (17.1) | 63 (14.5) | 129 (36.9) | 119 (34.2) | 5.7 |
| Exploration of uterine cavity and genital tract | 57 (7.3) | 48 (11.1) | 204 (58.4) | 190 (54.6) | 7.6 |

© 2019 Henriquez DDCA et al. *JAMA Network Open.*

**eTable 4, *continued*.** Characteristics of Women With Persistent Postpartum Hemorrhage for Sensitivity Analysis at 180 Minutes

| | Women, No. (%) | | | | |
|---|---|---|---|---|---|
| | **Overall cohort** *Characteristics at moment of diagnosing persistent postpartum hemorrhage* | | **Propensity score matched cohort** *Characteristics at moment of matching* | | |
| **Characteristic** | **No or later plasma transfusion[d] (n = 783)** | **Plasma transfusion within 180 minutes (n = 433)** | **No or later plasma transfusion[d,e] (n = 348)** | **Plasma transfusion within 180 minutes[e,f] (n = 348)** | **Standardized difference after propensity score matching (%)** |
| Intra-uterine balloon tamponade (Bakri) | 5 (0.6) | 4 (0.9) | 79 (22.6) | 74 (21.3) | 3.2 |
| **Hemostatic interventions** | | | | | |
| Tranexamic acid | 15 (1.9) | 21 (4.8) | 121 (34.8) | 118 (33.8) | 2.0 |
| Fibrinogen concentrate | 2 (0.3) | 5 (1.2) | 10 (2.9) | 12 (3.4) | 2.5 |
| Recombinant factor VIIa | - | - | - | - | - |
| **Transfusion[a]** | | | | | |
| Packed red blood cells | | | | | |
| 0 | 758 (96.8) | 389 (89.8) | 59 (17.0) | 60 (17.2) | reference |
| 1 | 10 (1.3) | 16 (3.7) | 69 (19.7) | 61 (17.4) | 5.8 |
| 2 | 9 (1.1) | 15 (3.5) | 142 (40.7) | 133 (38.2) | 5.1 |
| 3 | 3 (0.4) | 4 (0.9) | 43 (12.3) | 48 (13.9) | 4.6 |
| ≥4 | 3 (0.4) | 9 (2.1) | 36 (10.3) | 47 (13.3) | 9.4 |
| Platelets | | | | | 0.7 |
| ≥1 | 1 (0.1) | 5 (1.2) | 9 (2.6) | 9 (2.7) | |

[a]Covariate entered as a categorical variable in the propensity score model, with the first category as reference category. [b]includes genital tract trauma, placenta previa, placental abruption and congenital or acquired coagulation disorders. [c]Covariate entered as a continuous variable in the propensity score model. [d]'No or later plasma transfusion' includes women with no plasma transfusion and women with plasma transfusion at a later time point during hemorrage. [e]The proportion of women who have ondergone a time-dependent intervention increases during the course of postpartum hemorrhage, as an increasing amount of interventions will be performed in a single

© 2019 Henriquez DDCA et al. *JAMA Network Open.*

woman until cessation of the hemorrhage.[f]Numbers of women and proportions are averages derived from 10 imputed databases, and numbers of women were rounded to the nearest integer. Therefore, they may exceed the 'total' number of women or a proportion of 1, and the same number of women may correspond to different proportions.

© 2019 Henriquez DDCA et al. *JAMA Network Open.*

**eTable 5.** Sensitivity Analyses Excluding Pairs of Women With Cross-Overs From No or Later Plasma to Plasma Shortly After Matching

| Restriction on cross-over time interval | Average number of pairs of women | OR (95% confidence interval) |
|---|---|---|
| **Main analysis** | 114 | 1.09 (0.57-2.09) |
| **No cross-over within 15 min after matching** | 96 | 1.07 (0.51-2.23) |
| **No cross-over within 30 min after matching** | 79 | 1.09 (0.47-2.53) |
| **No cross-over within 45 min after matching** | 65 | 1.06 (0.41-2.73) |
| **No cross-over within 60 min after matching** | 59 | 1.13 (0.42-3.04) |

© 2019 Henriquez DDCA et al. *JAMA Network Open.*
